# State-of-the-art review: stress T1 mapping—technical considerations, pitfalls and emerging clinical applications

**DOI:** 10.1007/s10334-017-0649-5

**Published:** 2017-09-15

**Authors:** Stefan K. Piechnik, Stefan Neubauer, Vanessa M. Ferreira

**Affiliations:** Oxford Centre for Clinical Magnetic Resonance Research (OCMR), Division of Cardiovascular Medicine, Radcliffe Department of Medicine, University of Oxford, John Radcliffe Hospital, Oxford, OX3 9DU UK

**Keywords:** Cardiovascular magnetic resonance, Vascular reactivity, Stress, Tissue characterization, T1 mapping

## Abstract

In vivo mapping of the myocardial T1 relaxation time has recently attained wide clinical validation of its potential utility. In this review, we address the basic principles of the T1 mapping techniques, with particular attention to the emerging application of vasodilatory stress agents to interrogate the myocardial microvascular compartment, and differences between commonly used T1 mapping methods when applied in clinical practice.

## Introduction: what is T1 mapping?

T1 relaxation time, spin-lattice relaxation time, or simply T1, is the fundamental magnetic resonance property that describes the exponential recovery of the longitudinal component of magnetization (Mz) back towards its thermal equilibrium. In vivo, the recovery of Mz is complex, but characterizing the underlying processes with a single T1 value has shown promise as a biomarker [1]. The measured T1 is determined by intrinsic tissue properties, and the extrinsic environment, including surrounding structure and milieu, as well as software and hardware used to measure T1. Modern sequences allow direct generation of spatially resolved T1 relaxation maps. T1 mapping of tissues allows immediate assessment of their T1 values on a voxel-by-voxel basis as a method of direct quantitative tissue characterization. In general, each tissue type is expected to have a normal range of T1 values, deviation from which may indicate disease or a change in physiology.

## T1 mapping sequences

Myocardial T1 values measured in vivo depend on the chosen method, and are influenced by technical factors, such as magnetic field strength and pulse sequence design, and physiologic factors, including heart rate, temperature, age, gender, and disease [1]. The general design of T1 mapping sequences includes delivery of a pre-pulse and acquisition of multiple T1-weighted images to allow fitting of these signals to an exponential recovery curve. Common T1 mapping sequences used for cardiac T1 mapping are inversion recovery techniques [2–5], saturation recovery techniques [6], and mixed hybrid approaches [7].

**Fig. 1:**
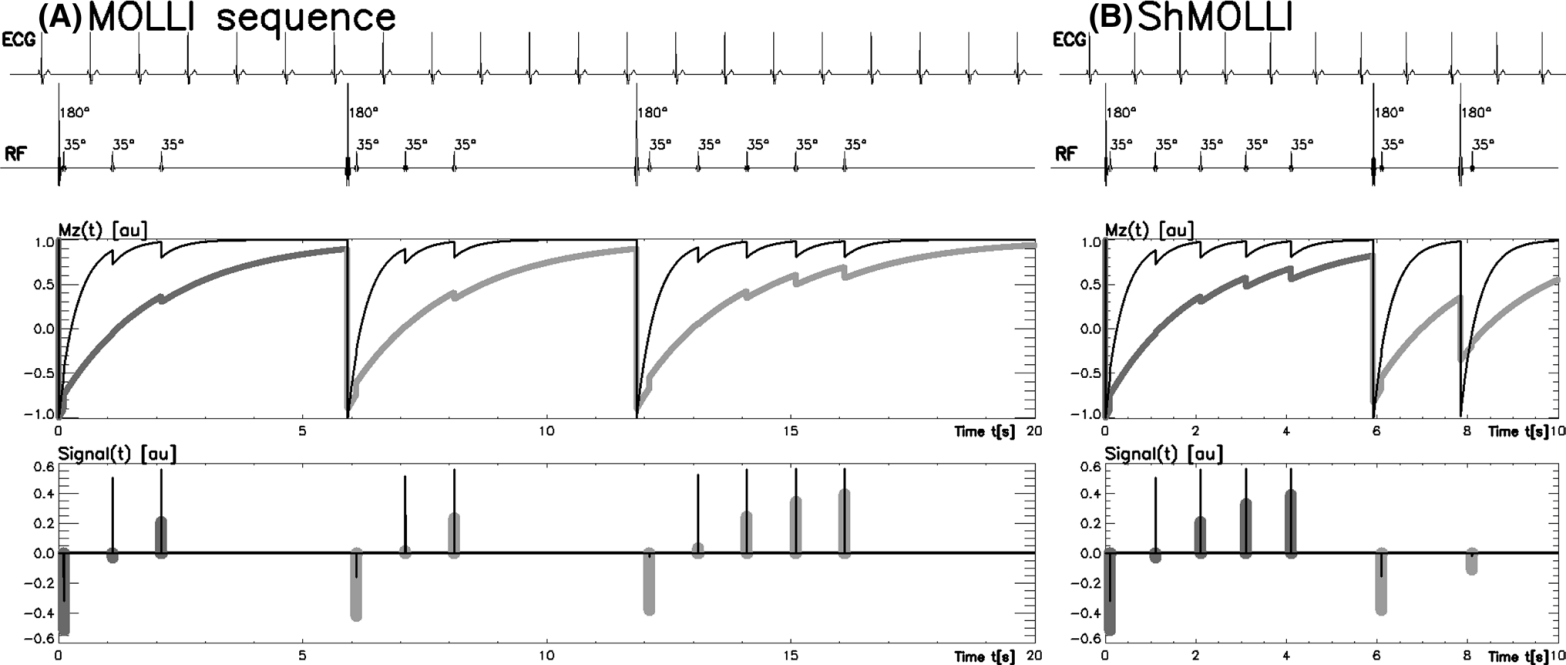
ECG-gated pulse sequence schemes for simulation of **a** MOLLI and **b** ShMOLLI at a heart rate of 60 bpm. SSFP readouts are simplified to a single 35° pulse each, presented at a constant delay time TD from each preceding R wave. The 180° inversion pulses are shifted depending on the inversion recovery (IR) number to achieve the desired first TI of 100, 180 and 260 ms in the consecutive IR experiments. The *plots* below represent the evolution of longitudinal magnetisation (Mz) for short T1 (400 ms, *thin lines*) and long T1 (2000 ms, *thick lines*). Note that long epochs free of signal acquisitions minimise the impact of incomplete Mz recoveries in MOLLI so that all acquired samples can be pooled together for T1 reconstruction. In ShMOLLI, the validity of additional signal samples from the second and third IR epochs is determined by progressive nonlinear estimation. As originally published by BioMed Central in Piechnik [3]

Current cardiac T1 mapping techniques evolved from the original Look-Locker spectroscopic method developed in 1970 [8], and provide a time-efficient approach for T1 mapping. As the living heart is a dynamic organ that contracts and relaxes, modification of the original scheme was needed to assure acquisition of sufficient information without sacrificing accuracy and clinical utility. The modified Look-Locker inversion recovery (MOLLI) was developed in 2004 [2] to address this issue by introducing intermittent image acquisition using electrocardiographic (ECG) gating to target a designated phase of the cardiac cycle, and then repeating the inversion experiments after a carefully optimized delay time to obtain adequate information to fit a single exponential T1 recovery curve (Fig. 1a). This sequence scheme significantly advanced T1 mapping for cardiac applications, allowing acquisition of a cardiac T1 map within a manageable 17-heartbeat-long breathold.

**Fig. 2:**
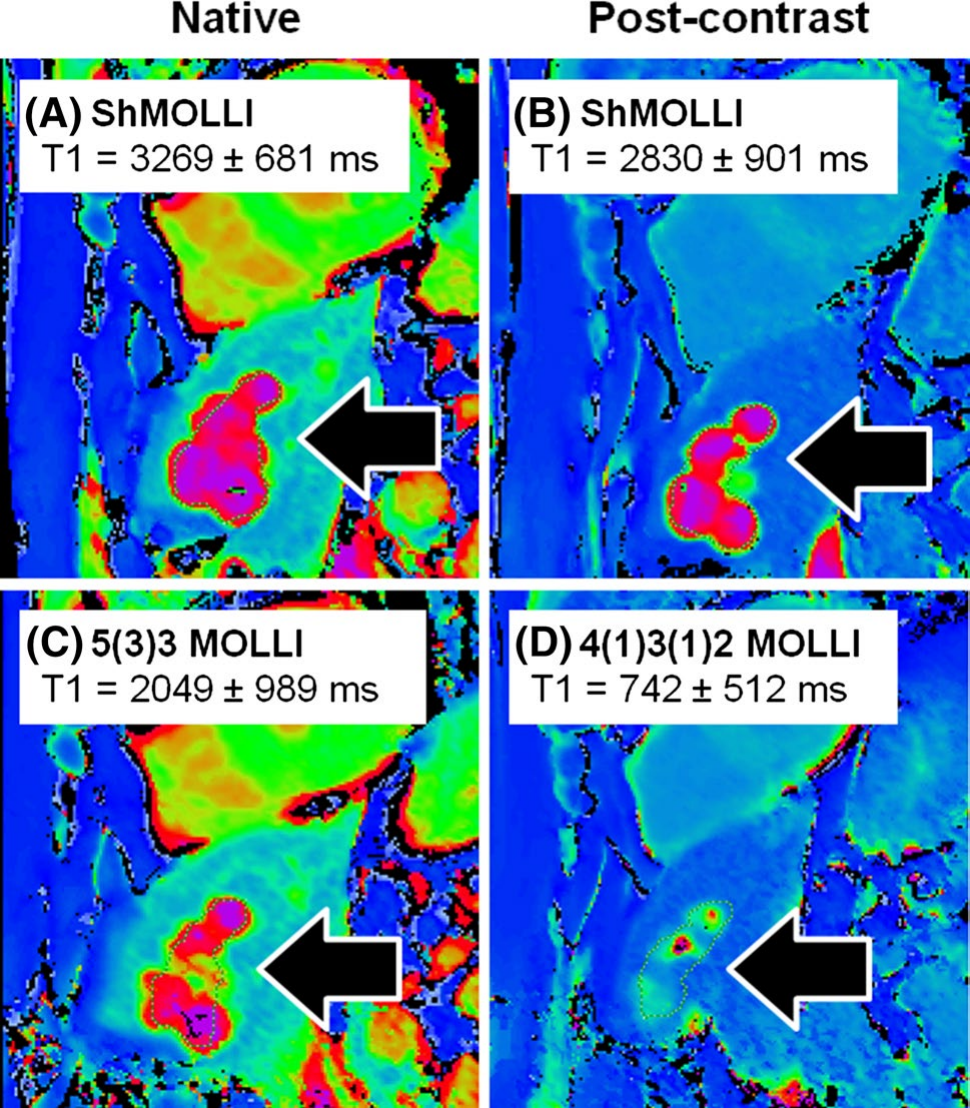
Characterizing tissues with very long T1 values using different T1 mapping techniques. Shown are T1 maps from a patient with a past history of breast cancer. Liver cysts (*black arrows*) observed with ShMOLLI retains the characteristic very long T1 both pre- (**a**) and post-gadolinium-based contrast due to its consistent performance over a wide range of heart rates and T1 values. In **c**, the 5(3)3 MOLLI variant pre-contrast T1 map shows ~30% lower T1 in the liver cysts, consistent with the back-loaded 11-heartbeart MOLLI 3(3)5 variant [4]. **d** Post-contrast T1 map using the 4(1)3(1)2 MOLLI variant dedicated for post-contrast applications suffers substantial underestimation of cystic T1 by >70%. Comparing **c** and **d**, cystic lesions may appear to take up gadolinium-based contrast agents (GCBAs), which may suggest a tumour with communication to the vasculature, rather than what would be expected for a cyst. T1 is quoted for manual regions of interests drawn within the cysts. *Colour tables* are identical for *all panels* shown, as in Siemens ShMOLLI distributions for ease of comparisons

The shortened modified Look-Locker inversion recovery (ShMOLLI 2010) [3] (Fig. 1b) further addressed several limitations of MOLLI-based cardiac T1 mapping towards practical clinical applications, and has been extensively validated clinically over the past 7 years [9–29]. In particular, advantages of ShMOLLI include:

1. Short breath-holds: it significantly shortened the breath-hold time to 9 heartbeats (usually around 10 s) per T1 map, rendering imaging time easier for sicker patients to cope with [3].
2. Heart rate independence: it eliminated heart rate dependency characteristic of other MOLLI-based techniques and variants, and is able to cope with tachyarrhythmias, such as rapid atrial fibrillation, frequent ectopic beats, and sinus tachycardia; this is particularly relevant for performing mapping during dynamic heart rate changes, such as for stress applications [3, 14, 27–29].
3. Flexibility: ShMOLLI is a one-for-all technique for a wide range of T1. In particular, it estimates long T1s without the progressive heart rate-dependent underestimation typical of MOLLI [3]. This is relevant for tissues such as blood (in the range of up to 2000 ms) and fluids such as in pericardial effusions, cysts, and cerebrospinal fluid (in the range of 3000–4000 ms). This feature is also important for assessing edematous tissues, and extracellular volume (ECV) where blood T1 is required. Other novel applications include characterization of masses (e.g. differentiating cysts from solid tumors) [30, 31], and splenic T1 to determine stress adequacy, which requires a T1 mapping sequence that can handle both long T1 values and in dynamic stress conditions [10, 11, 29, 32].
4. Practicality: ShMOLLI is able to simultaneously estimate short and long T1 pixels in the same image without requiring separate sequence sampling schemes for pre- and post-contrast T1 applications [33]; this makes it highly convenient in the practical workflow for ECV applications. It also allows post-contrast characterization of masses to determine gadolinium uptake, without misclassifying a cyst as a mass that appears to take up gadolinium contrast agents, which may, for example, suggest a vascular tumour on post-contrast T1 maps (Fig. 2).

There are other short MOLLI variants that have been developed also aimed at shortening imaging times [4, 33]. Currently, MOLLI-based sequences are the most commonly used and validated, although saturation-recovery single-shot acquisition (SASHA, SmarT1Map) sequences have attracted much attention due to acceptable short imaging times, nominal lack of heart rate dependency and excellent accuracy in estimating myocardial T1 times shown in simulation and in phantoms [34, 35]. Hybrid approaches that combine saturation and inversion pulses are also available as emerging techniques for cardiac applications [7].

## What do T1 measurements bring to clinical practice?

Myocardial T1 mapping methods can be used for native (or pre-contrast) T1 mapping, post-contrast T1 mapping, and ECV mapping (detailed review may be accessed elsewhere [36]). Briefly, native (pre-contrast) T1 reflects a composite signal from both the intracellular (predominantly myocytes) and extracellular spaces (which includes the interstitial and intravascular compartments). T1 predominantly detects free water, and increased free water content in tissue, such as edema or water collecting in expanded interstitial spaces. T1 does not directly detect collagen fibers, but predominantly the accumulation of water around fibrotic tissue which typically prolongs native T1 relaxation times and is responsible for the strong indirect links to areas of fibrosis reported in the literature. Processes that are known to lower T1 times include significant iron and fat content [26, 37, 38], as well as contrast agents, particularly gadolinium. Isolated, single time-point post-contrast T1 mapping is currently not preferred to estimating the ECV, due to strong dependencies on the timing and dose of contrast administered, and other confounding factors [1]. Instead, ECV may be quantified non-invasively using pre- and post-contrast T1 maps to obtain pre- and post-contrast myocardial and blood T1 values, adjusting for the hematocrit.

**Fig. 3:**
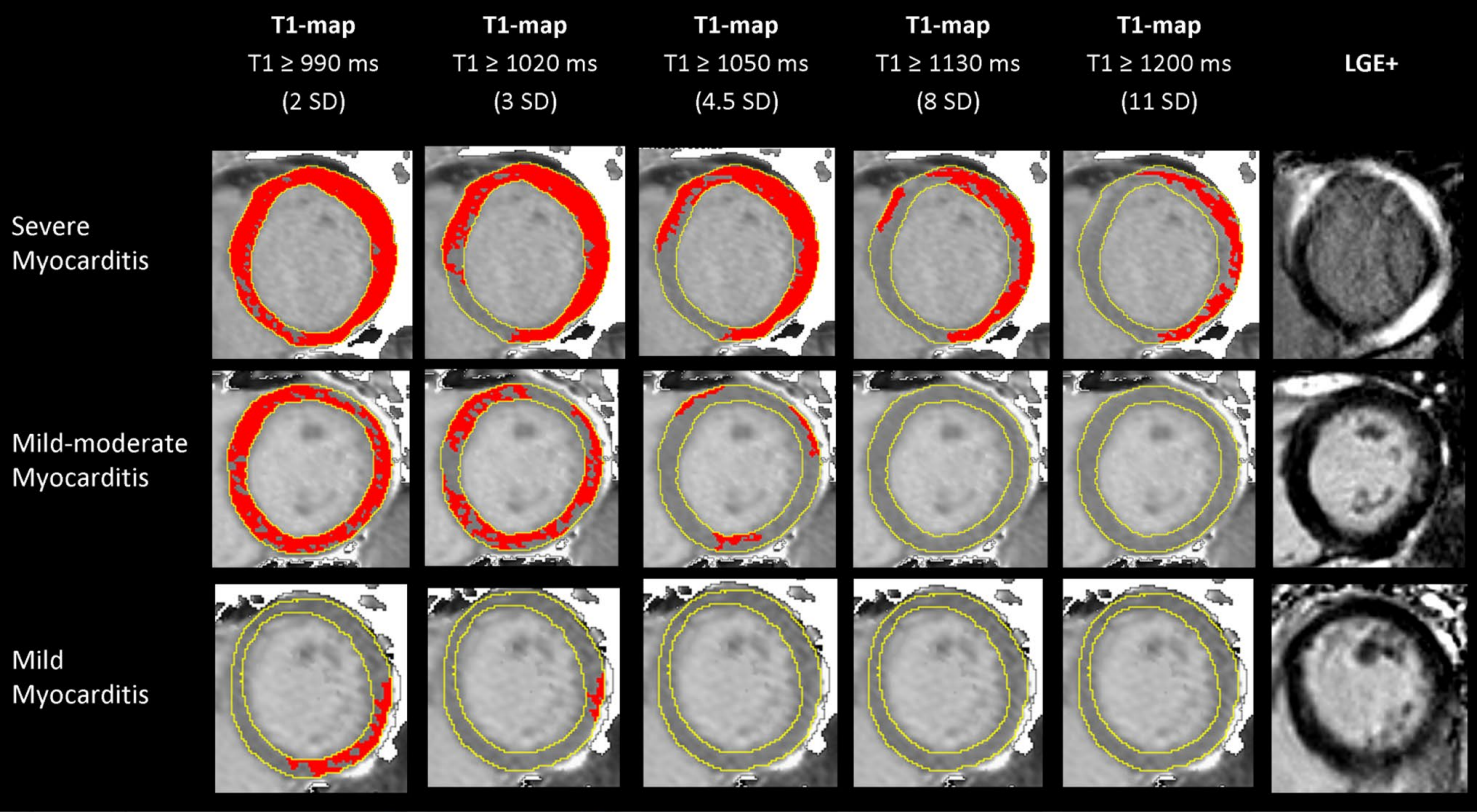
T1 maps using incremental thresholds demonstrate the predominantly non-ischaemic pattern of injury across a spectrum of acute myocarditis. *Red* indicates areas of myocardium with a T1 value above the stated threshold of at least 40 mm^2^ in contiguous area. A T1 threshold of 990 ms was previously validated for the detection of acute myocardial oedema; other thresholds were selected for illustrative purposes. As originally published by Biomed Central in Ferreira et al. [13]

It is important to emphasize that T1 biomarkers are non-specific and may deviate from their normal ranges due to a variety of causes. In particular, T1 and ECV may act as a surrogate for interstitial fibrosis only if other confounding factors of increased T1 or ECV—including edema, inflammation, amyloidosis that expand the interstitial space, and ischemia—have been excluded [1, 27, 28]. Current evidence demonstrates that native myocardial T1 values can be measured within a tight normal range, with clinically relevant sensitivity to changes in a wide range of cardiac diseases [36, 39, 40]. T1 maps can be displayed using color scales or threshold-based overlay masks to highlight tissue differences and aid visual interpretation [11, 13, 21, 31, 41, 42] (Fig. 3). Native T1 maps allow differentiation of an increasing range of tissue types without the need for gadolinium-based contrast agents (GBCA).

## Principle of gadolinium-free T1 mapping to assess the coronary vascular compartment

**Fig. 4:**
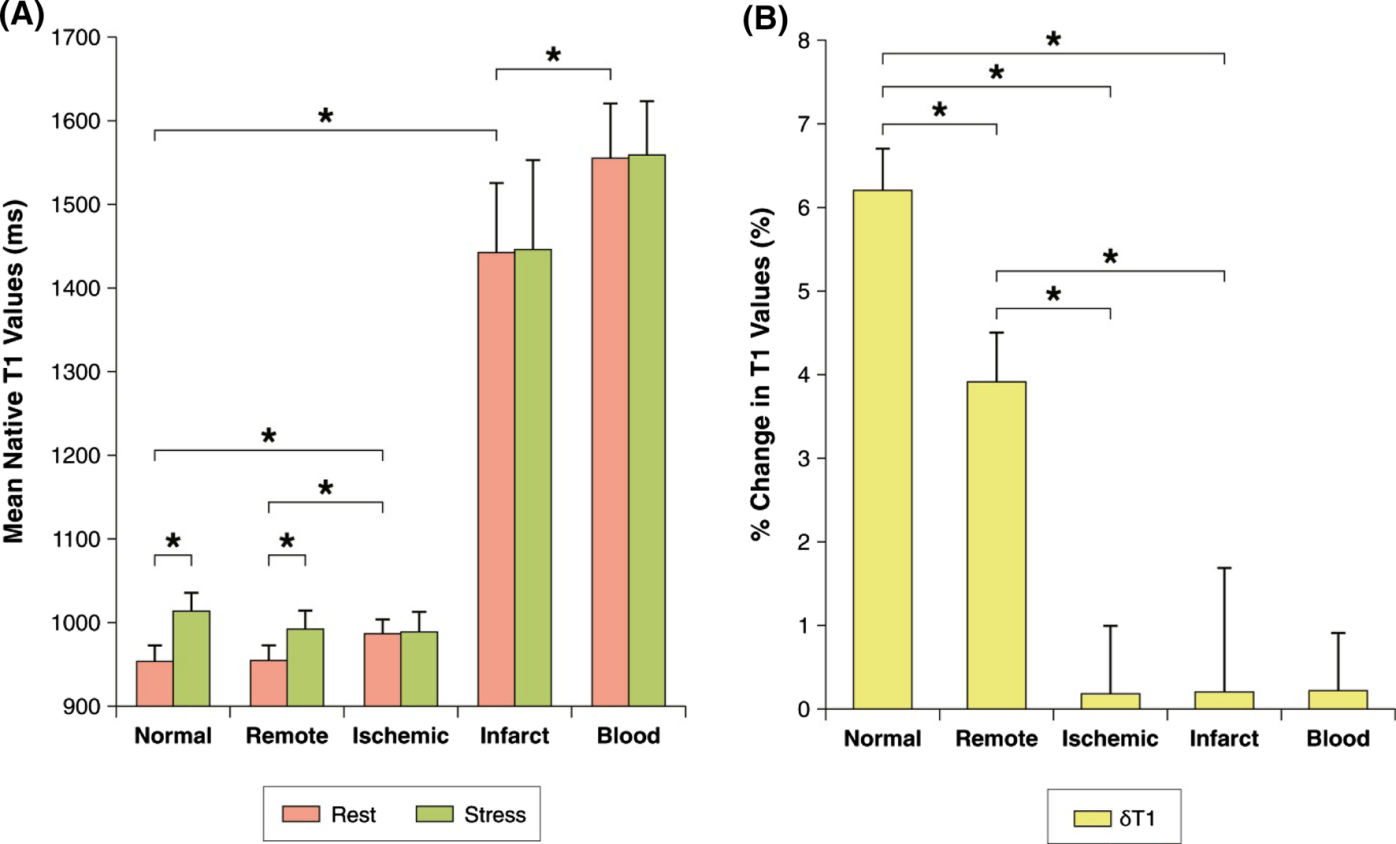
Myocardial T1 at rest and during adenosine stress at 1.5 T. **a** T1 values at rest in normal and remote tissue were similar and significantly lower than in ischemic regions. Infarct T1 was the highest of all myocardial tissue, but lower than the reference left ventricular blood pool of patients. During adenosine stress, normal and remote myocardial T1 increased significantly from baseline, while T1 in ischemic and infarcted regions remained relatively unchanged. **b** Relative T1 reactivity (δT1) in the patient’s remote myocardium was significantly blunted compared to normal, and completely abolished in ischemic and infarcted regions. All data indicate mean ± 1 SD. *p < 0.05. As originally published by Elsevier in Liu [28]

Myocardial blood volume (MBV) constitutes ~10% of the total myocardial volume at rest [43], and may increase two-fold during coronary vasodilatory stress [44, 45]. In healthy individuals with normal myocardium and coronary arteries, there is significant coronary vasodilatory reserve, which can be interrogated by administration of adenosine vasodilatory stress [46]. Coronary vasodilation augments both coronary blood flow as well as intramyocardial blood volume [45]. Since native blood T1 is much longer than native myocardial T1, blood T1 is expected to increase the measured myocardial T1 through its partial volume effects [9]. This has been shown in normal volunteers who exhibit a 6% increase in myocardial T1 with narrow normal ranges during adenosine vasodilator stress, using the heart rate-independent ShMOLLI method (6.2 ± 0.5% at 1.5 T; 6.3 ± 1.1% at 3 T) [28] (Fig. 4).

## Stress and rest T1 mapping in coronary artery disease (CAD)

Stress T1 mapping has obvious potential applications in patients with CAD and ischemic heart disease [28, 47]. Liu et al. [28] demonstrated that, in normal myocardium, the resting T1 is normal, with a 6% rise during vasodilatory stress. In chronic infarcted myocardium, the resting T1 is typically significantly elevated compared to normal myocardium, with no change in T1 during stress (Fig. 4). In ischaemic myocardium subtended by a significant coronary stenosis, there is compensatory downstream coronary vasodilation even at rest; this is detectable as mildly elevated resting myocardial T1 values, but do not show further coronary vasodilatory response during stress, and, thus, no change in stress myocardial T1. Adenosine stress and rest T1 mapping may be used to distinguish normal, infarcted, and ischaemic myocardium, without the need for GCBA, due to their distinctive rest and stress T1 profiles [28] (Fig. 4).

## Stress and rest T1 mapping in patients without obstructive CAD

**Fig. 5:**
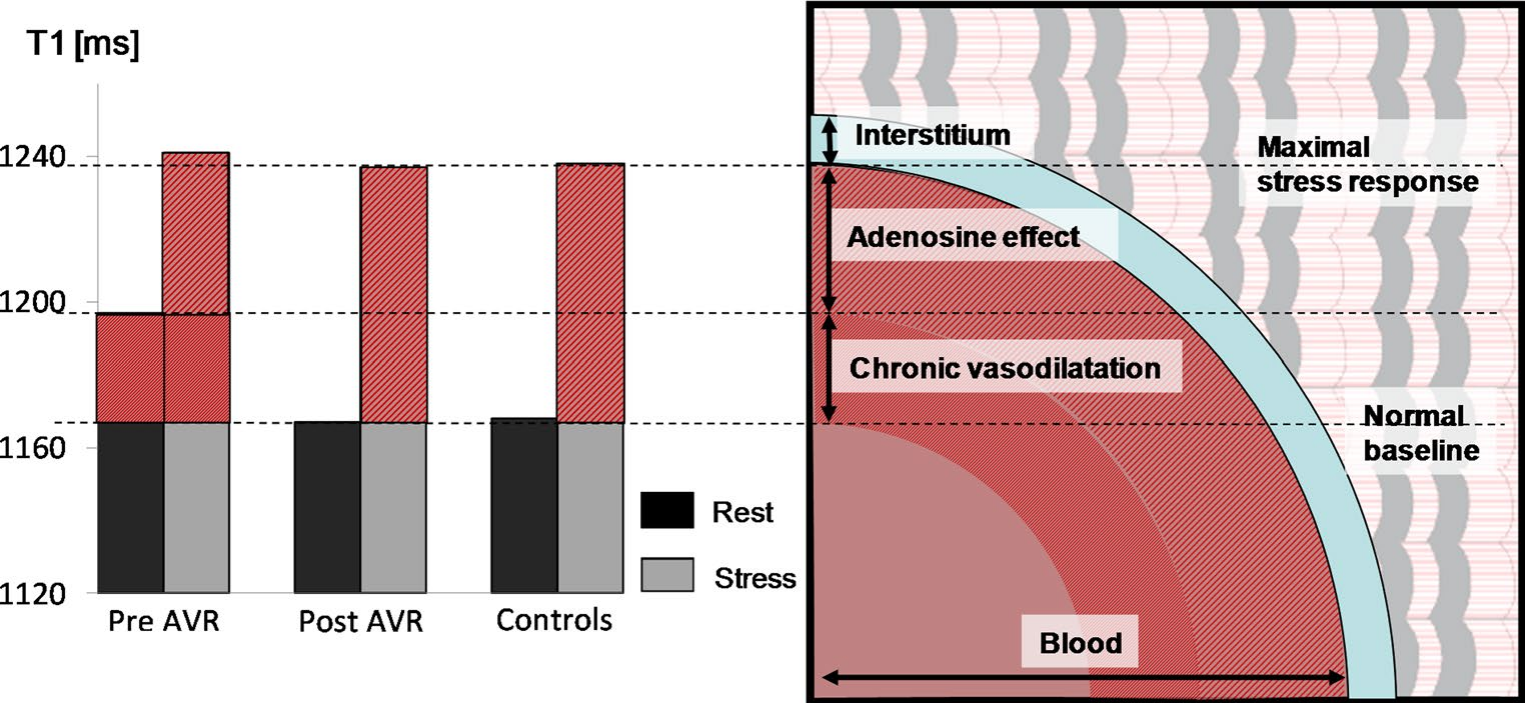
Proposed myocardial water compartments in aortic stenosis. Proposed changes in myocardial water compartments at rest and stress in patients with aortic stenosis pre and post AVR, and controls (*left*). The T1 response to adenosine was mainly contributed to by vascular responses instead of interstitial space expansion which may be negligible. Note that T1 and volumes from vascular cross-sections are for qualitative comparison only and not to scale. As originally published by BioMed Central in Mahmod et al. [27]

Adenosine stress and rest T1 mapping may also be used to assess coronary vasodilatory reserve in patients without obstructive CAD. For instance, in patients with type 2 diabetes without obstructive CAD, early data have shown blunted stress T1 responses compared to controls, which may reflect microvascular dysfunction [48, 49], and is a subject of further investigation. In patients with severe aortic stenosis but no obstructive CAD on invasive angiography, the increased demands of the pressure-overloaded and hypertrophied myocardium are accompanied by increased resting coronary blood flow and vasodilation [50–52]. This is detectable as elevated resting myocardial T1, but achieving the same maximal adenosine stress T1 response when compared to normal controls [27]. This impaired stress T1 response normalizes 7 months after relief of the pressure overload with aortic valve replacement [27] (Fig. 5). This finding supports the notion that, in severe aortic stenosis, increased resting myocardial T1 may mainly reflect changes in the intravascular compartment, rather than solely from diffuse myocardial fibrosis in the interstitial compartment as previously believed, although these two processes likely co-exist in this disease model. Other investigators have explored stress T1 mapping as a surrogate marker for myocardial blood volume change in heart transplant recipients [48, 49]. We believe that stress T1 mapping holds promise for assessing coronary microvascular function and vasodilatory reserve in a number of cardiomyopathies as emerging clinical applications.

## Splenic T1 mapping: a novel surrogate marker for adequate adenosine stress

**Fig. 6:**
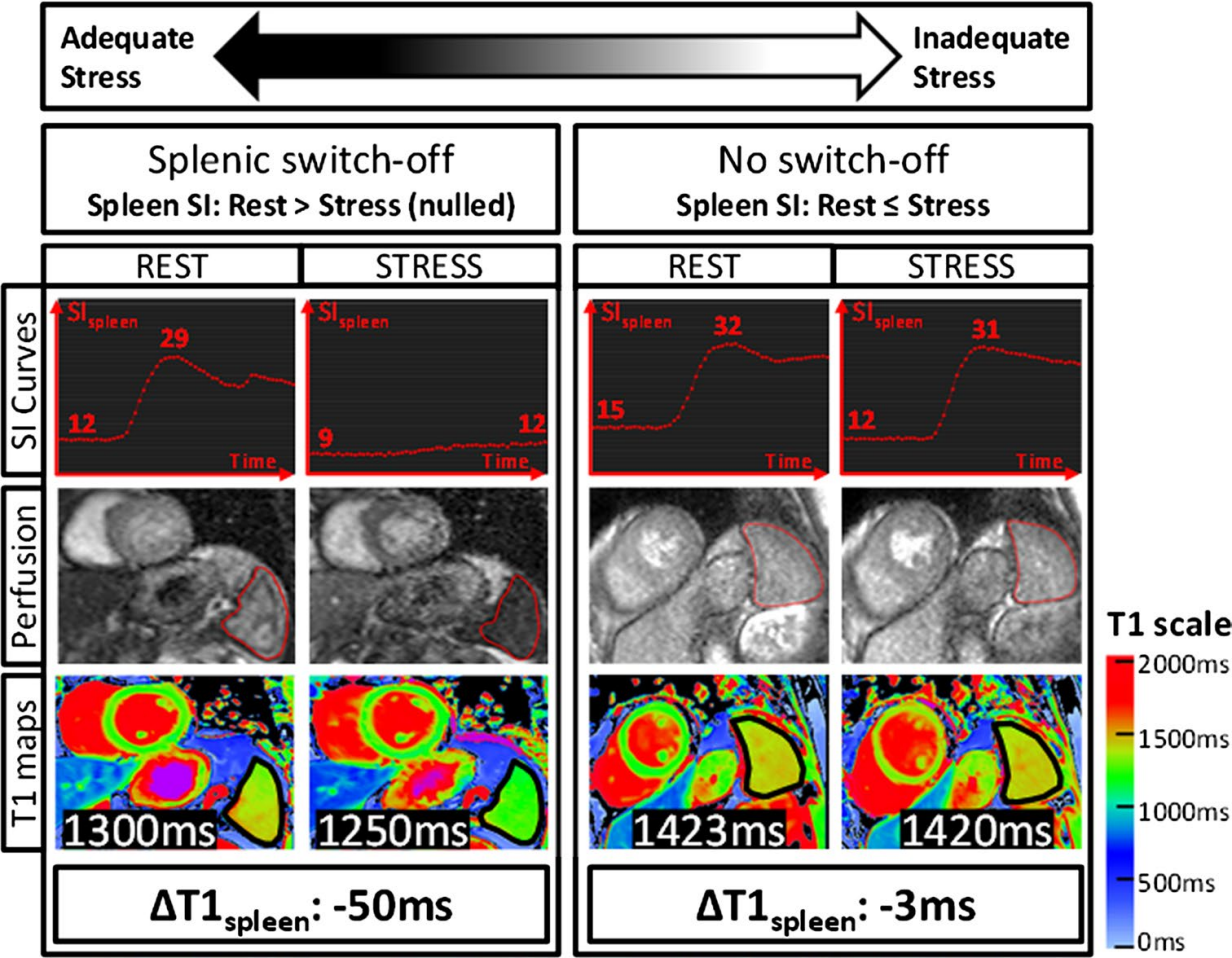
Representative stress and rest splenic first-pass gadolinium perfusion and native T1 maps. Signal intensity (SI) *curves* represent splenic perfusion SI (*y*-*axis*, arbitrary units) over time (*x*-*axis*, 50–60 s). The maximum and minimum SI_spleen_ are as indicated. Splenic regions of interests on perfusion images and T1 maps are outlined in *red* and *black*, respectively. Mean native T1_spleen_ and stress changes (ΔT1 _spleen_) are as labelled. 3 T images were used for illustration (observed ΔT1 _spleen_ and ΔSI _spleen_ are field strength-independent). As originally published by BioMed Central in Liu et al. [29] (color figure online)

Stress adequacy is an integral component of the cardiac stress examination, which may impact on the diagnostic confidence, especially for ruling out significant obstructive CAD. Recently, stress T1 mapping of the spleen has been shown to be promising novel invention for assessing adenosine stress adequacy before stress perfusion clinical magnetic resonance (CMR) imaging [29] (Fig. 6). Whilst adenosine stress induces vasodilation in the coronary arteries, it simultaneously induces vasoconstriction in the spleen. This is manifest as the “splenic switch-off sign”, which can be seen on nuclear stress perfusion [53] as well as CMR gadolinium-based perfusion imaging, and may serve as a marker for adenosine stress adequacy [54]. On CMR perfusion images, the spleen is typically visible in the field of view, and during peak adenosine stress, the spleen appears dark (“switch-off”) compared to rest perfusion images when the spleen appears bright (as it takes up GBCA). The lack of “splenic switch-off” has been observed in more false negative perfusion CMR scans when compared to invasive coronary angiography in detecting significant CAD [54]. One limitation of the gadolinium-based “splenic switch-off” sign is that to visualize this interesting phenomenon, GBCA would have already been administered for first-pass perfusion imaging, and does not leave an opportunity to optimize the adenosine stress protocol on the fly. Splenic T1 mapping, on the other hand, does not require GBCA, and splenic vasoconstriction associated with adenosine stress significantly decreases splenic T1 values, which can be conveniently detected on stress T1 maps typically without additional planning [29]. This provides a pre-emptive opportunity to increase and/or prolong adenosine administration to achieve adequate adenosine stress before acquiring stress images to increase diagnostic confidence. Splenic T1 mapping is undergoing further validation for this indication.

## Pitfalls of stress T1 mapping

### The impact of the chosen T1 mapping technique on rest and stress T1

It is widely recognized that T1 mapping techniques, even within a method family like MOLLI-based sequences, have different properties and diverging norms [1, 55]. The impact of this issue is particularly apparent for stress T1 applications, as illustrated by two recent studies that used different MOLLI techniques to perform adenosine stress T1 mapping: Liu et al. [28] performed stress T1 mapping using ShMOLLI to study normal volunteers compared to patients with CAD, while Kuijpers et al. used MOLLI 5(3)3 stress T1 mapping to study patients who had normal findings on conventional CMR (served as controls) compared to CAD patients. Liu et al. observed a normal stress T1 response of 6.2 ± 0.5% at 1.5 T and 6.3 ± 1.1% at 3 T [28], while Kuijpers et al. obtained a lower stress T1 response and larger standard deviations of 4.3  ±  2.8% in controls at 1.5 T [47]. In CAD patients with remote myocardium, Liu et al. noted a blunted T1 response of 3.9 ± 0.6%, while Kuijpers et al. reported lower averages of 2.6  ± 3.4%. Similarly, another group of investigators reported that MOLLI 5(3)3 achieved a stress T1 response of 3.3% (1.5 T) and 4.4% (3 T) [47, 56]. Recently, stress T1 responses using regadenoson showed reactivity similar to those previously reported after adenosine administration [57].

It is encouraging that the stress T1 response can be elicited using more than one T1 mapping technique by independent groups of investigators and with different stress agents. At the same time, the fact that the ShMOLLI stress T1 response is larger by >40% than using MOLLI 5(3)3 based on the published numbers above deserves attention and discussion. Conventionally, CMR methods that compare images before and after an intervention (such as administration of a stress agent or GBCA, as in perfusion imaging and ECV mapping) within the same subject in a single scan session may improve inter-individual and inter-center consistencies of the imaging biomarker. However, the same cannot be said for stress T1 mapping based on current limited evidence. In the two studies cited above [28, 46], the resting T1 (955 ± 17 ms in Liu et al. and T1_rest_ 977  ±  40 ms in Kuijpers et al.) amount to only a ~2% difference between these two techniques. In contrast, the inter-methodological differences in stress T1 responses differ by ~40% (approximately 6 vs. 4%, respectively)—i.e., a 20-fold worse agreement than for resting T1 values. Potential reasons for discrepancies in the stress T1 response between these two techniques may include factors such as selection of patients (with normal findings) as controls in the MOLLI study and control age differences, adenosine stress duration, adequacy, and maximal heart rate achieved; however, the technical differences between the ShMOLLI and MOLLI 5(3)3 T1 mapping techniques and their impact on the stress T1 response also require further consideration, as discussed below. Early standardization may be even more important for stress applications than for native resting T1.

### The impact of heart rate variation on stress T1 mapping

**Fig. 7:**
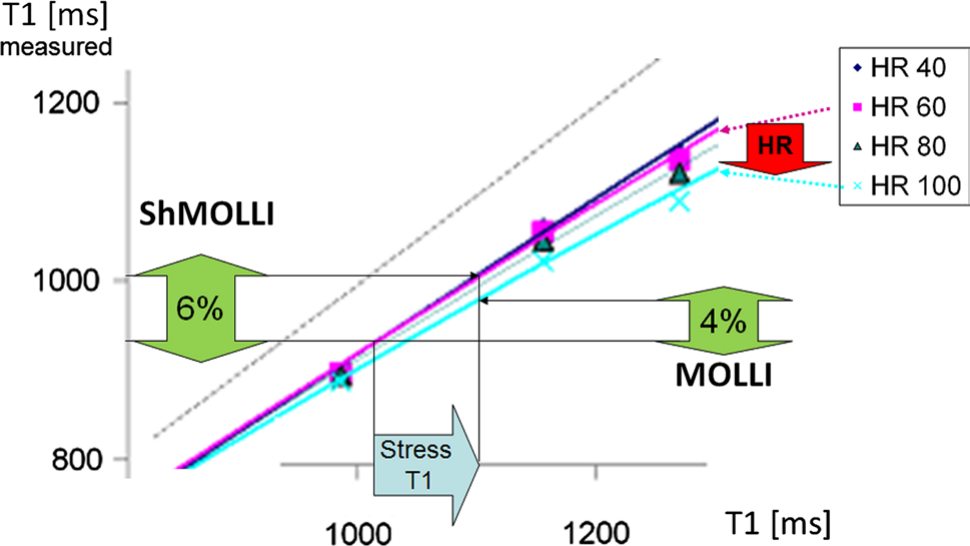
Mechanism for the impact of heart rate sensitivity on the measured stress T1 responses using MOLLI variants. MOLLIs generally underestimate T1, hence all *coloured lines* are under the *unity line* (*grey dotted*). ShMOLLI has no heart rate (HR) dependence, and behaves like the HR 40 (*dark blue*) line across the HR range of 40–100 beats per minute. As a result, when myocardial T1 increases during vasodilatory stress (*solid blue arrow*, *x-axis*), this corresponds to just moving along a single linear relationship (*dark blue* HR 40), and preserves the relative size of the T1 response (6%). The MOLLI 3(3)5 variant [4] illustrated here is HR dependent. Thus, when myocardial T1 increases during vasodilatory stress, the transition involves simultaneous switching between HR-dependent relationships (*red arrow* “HR”). This results in a lower ~4% stress T1 response using the MOLLI 3(3)5 variant. Adapted from Fig. 2 originally published by BioMed Central in Lee et al. [4] (color figure online)

ShMOLLI, with a sampling scheme of 5(1)1(1)1, is “front-loaded” by acquiring most samples upfront and is heart rate-independent due to its in-built conditional reconstruction algorithm [3, 28]. This in contrast to earlier MOLLI techniques [58], which tend to be “back-loaded”, with most samples acquired at the end of the sampling scheme, such as the classic MOLLI 3(3)3(3)5 design [2]. The MOLLI 5(3)3 variant aimed to reduce heart rate sensitivity also by front-loading [59] but does not ultimately eliminate it, as all data are used to fit to a single model, regardless of whether recovery epochs are adequately long or not (see Fig. 2). Given that stress T1 responses are relatively small, even a slight degree of residual heart rate sensitivity in the myocardial T1 range can impact on the observed stress T1 reactivity. Figure 7 illustrates the mechanism of stress T1 underestimation using 11-heart-beat 3(3)5 MOLLI due to heart rate dependency based on data published by an independent group of investigators [4]. The most recent MOLLI variant, using a sampling scheme of 5s(3s)3s, has been proposed to further reduce heart rate sensitivity [60]. However, even within the relatively limited range of T1 (0–1200 ms) validated for this technique (further details in Fig. 9 in [60]), the T1 and heart rate dependence are still evident. It remains unclear what proportion of the significant T1 underestimation seen in wider T1 ranges beyond 1200 ms, as reported by other studies for classic MOLLI [3, 4], may remain for MOLLI 5s(3s)3s.

SASHA T1 mapping is heart rate independent under optimal conditions, although there have been no reports of its application during dynamic stress thus far. It remains to be tested in practice by further comparisons whether the lower signal-to-noise ratio (SNR) known for this technique and whether imperfect saturation conditions may introduce confounds based on the known error dependencies (shown in Figs. 2, 3, 4 in the original paper [6]).

### Factors other than heart rate that impact on stress T1 mapping

There are factors other than heart rate that may impact on the stress T1 response for a T1 mapping technique, which include T1 sensitivities to T2, magnetization transfer (MT) effects, and breath-hold duration and motion during stress conditions.

With regard to T2 sensitivities and MT effects, these properties that confer MOLLI-based techniques their recognized sensitivity to detecting disease [35] are likely to enhance their sensitivity to the stress T1 response elicited by ShMOLLI. Assuming that the underlying mechanism of the stress T1 response is mainly related to an increase in blood volume, the increased water content will directly affect MT and T2 to synergistically increase the measured T1. Further, the stress ShMOLLI-T1 response is likely to be enhanced by residual sensitivity to T2 elevations due to underlying BOLD (blood oxygenation level dependent) effects. The surplus BOLD response is characteristic of normal vascular reactivity [61–63] and will also accentuate the contrast to pathological changes. Conversely, while stress T1 mapping has not been reported using saturation-recovery techniques, the lack of MT and T2 dependencies will likely diminish the stress T1 response, especially given the higher variability (noise) typically seen in T1 estimation using saturation-recovery methods [6, 35, 64]. Recently improved inversion pulses in recent MOLLI variants target the accuracy of T1 by reducing T2 and MT sensitivities, which may be paradoxically detrimental to their sensitivity to detect stress T1 responses [35].

For the more recent MOLLI variants that use sampling schemes measured in seconds (rather than in heartbeats) [60], at increased heart rates, there will be more image acquisitions per inversion recovery experiment, and more energy deposited into bound proton pools. For example, MOLLI 5s(3s)3s would deploy as MOLLI 5(3)3 at a heart rate of 60 beats per minute, but at 120 beats per minute, MOLLI 10(6)6 would be deployed, with twice as many readouts. Thus, MT sensitivity between the actual variants deployed at rest and during stress is likely to differ significantly. What happens exactly is largely academic, as its clinical application is likely to be more impacted by the breath-hold requirements. Human subjects undergoing dynamic stress using the 5s(3s)3s sampling scheme would need to hold their breath typically for 12–14 s, longer than classic MOLLI under the same stress conditions. Kuijpers et al. [47] had reported substantial motion artefacts using MOLLI 5(3)3 for stress T1 mapping, and these are likely to worsen using MOLLI 5s(3s)3s due to longer breath-hold requirements. Recent studies agreed that motion remains a substantial concern for MOLLI acquisitions for stress applications, which could not be overcome by inline MOCO [47, 56].

It would be difficult to explain the observed dependencies quantitatively by simple partial-volume relaxivity summation. We draw attention to the complex spectrum of blood T1, T2, and volumetric reactivity between various vascular compartments in the brain (details in Figs. 11–14 in [61], supplemental material). Briefly, these demonstrate very significant differences in baseline values and stress reactivity of T1 and T2 of blood in various vascular compartments in the brain, with a disparately small blood volume attributed to the vaso-reactive arterial component when compared to the capillary and venous bed. These effects in the brain have been studied in response to CO_2_ administration, but not with adenosine or the dynamically changing tissue stress that occurs with each heart beat. These factors are important, as the dynamics of compartmental volume redistribution depend on time scales and the types of stimuli in the brain [65]. Given the challenges to gather similar data for the heart, ultimately, the diagnostic performance of a method to study the heart during stress conditions will boil down to clinical evidence and independent head-to-head comparisons in clinical practice [64]. Computer simulations and phantom experiments, while helpful as initial guides to assess a new method, may not fully replicate or account for factors encountered in the in vivo environment [64], especially in a complex and dynamic organ like the human heart.

## Future directions and implications

Stress and rest T1 mapping is a novel technique with potential to assess ischaemia, coronary vasodilatory reserve, and the health of the micro-coronary circulation, without the need for GCBA. T1 mapping is a nascent field, and the exact biological mechanisms of native and stress T1 signals in various conditions have not been fully elucidated. The effects of other modalities of stress, including exercise and pharmacological agents, as well as other modulators of vascular reactivity on T1 may be explored to fully determine its clinical applicability. Stress T1 mapping is an active area of scientific development, including validation against quantitative perfusion measures, invasive coronary measurements and diagnostic performance in a variety of cardiac conditions [55, 66–70]. Over time, collective evidence will allow better understanding of the mechanisms for the observed changes for this emerging technique and its clinical utility in a wider patient population, including those with contraindications to GBCA.
